# Patients with Diabetes Complicated by Peripheral Artery Disease: the Current State of Knowledge on Physiotherapy Interventions

**DOI:** 10.1155/2021/5122494

**Published:** 2021-05-10

**Authors:** Katarzyna Hap, Karolina Biernat, Grzegorz Konieczny

**Affiliations:** ^1^Department and Division of Medical Rehabilitation, Wroclaw Medical University, Wroclaw, Poland; ^2^Faculty of Health Sciences and Physical Education, Witelon State University of Applied Sciences in Legnica, Poland

## Abstract

Diabetes mellitus (DM) is one of the major public health problems that account for morbidity, mortality, and disability worldwide. The presence of DM increases the risk of peripheral artery disease (PAD), as well as accelerates its course, making these patients more susceptible to ischemic events and impaired functional status. Unfortunately, alternative treatments for vascular complications in diabetes are poorly researched. Physiotherapy (kinesitherapy combined with different physical therapy agents) in individuals with DM and coexisting PAD may offer an important complementary therapy alternative. Early therapeutic measures can significantly improve patient outcomes, reduce cardiovascular risk, and improve daily life quality. The article provides an update on the current state of knowledge on physiotherapy interventions in the course of DM in patients with coexisting PAD.

## 1. Introduction

Atherosclerosis is responsible for chronic ischemia of the extremities, mainly the lower ones, in 20–30% of the population over 50 years of age [1]. Peripheral artery disease (PAD) affects approximately 202 million people [2], and progressive atherosclerotic lesions, leading to stenosis and occlusion in the arteries, are the cause of the claudication, which reduces patients' quality of life, and ultimately may be the cause of amputation [1, 3].

Lower extremity atherosclerosis is also one of the major chronic complications of diabetes mellitus (DM), a condition that has become an epidemic of the 20th and 21st centuries. Based on the World Health Organization (WHO) report [4], it is a known fact that the number of people with diabetes is steadily increasing worldwide, and experts estimate that by 2045 there will be at least 629 million people living with the disease [4]. It also means an increase in the number of people affected by its chronic complications. DM is the second (after nicotinism) most significant risk factor for PAD, and patients with diabetes have as much as 2–4 times greater risk of developing atherosclerosis [5, 6]. This risk is influenced by glycemic control—every glycated hemoglobin (HbA1c) increase by 1% is associated with a 28% increase in the relative risk for manifest PAD [6]. In addition, the higher risk of atherosclerosis in patients with type 2 diabetes mellitus (T2DM) is due to numerous metabolic disorders coexisting with hyperglycemia, e.g., unfavorable lipoprotein profile or hypertension. Therefore, all these cardiovascular risk factors should be intently monitored and corrected to prevent the development of major adverse ischemic limb events [7, 8].

Furthermore, both the prevalence of atherosclerosis and T2DM increase with age, which is of epidemiological significance in aging populations. The cooccurrence of lower limb atherosclerosis and diabetes is particularly harmful to patients because vascular lesions appear earlier, are more diffuse, and usually distally located, making their surgical treatment less effective [9–12]. Critical limb ischemia (CLI) is much more frequent in patients with DM who have an almost twice higher risk of amputations compared to people without diabetes [11, 13, 14]. Also, the mortality rate after amputations in this group is very high at 50–74% in 5-year all-cause mortality [15].

Concomitant peripheral neuropathy, one of the chronic microcomplications of diabetes, may mask typical limb ischemia symptoms for years, promoting disease progression and delaying proper treatment [9]. The project published by Nichols suggests that diabetic peripheral neuropathy (DPN) and PAD are strongly related and that DPN may often precede PAD [16]. On the other hand, arteria stenosis aggravates DPN [16]; thus, the relation seems to be bidirectional.

The first, noncharacteristic symptoms of lower limb ischemia, such as cramps, numbness, or hypersensitivity to cold (Fontaine stage I) [17], can be confused with sensory neuropathy. The most common symptom that patients present is claudication (Fontaine classification stage II). This unpleasant symptom can also be masked by concomitant neuropathy or confused with musculoskeletal problems, which are also often found in the obese population of patients with T2DM. As a result, these patients often visit doctors when the disease is advanced and rest pain and/or necrosis (Fontaine stage III and IV, respectively) appears, which requires surgical intervention and is the cause of the most common, nontraumatic amputations in the world [17–19]. PAD prevalence in DM patients is difficult to accurately estimate because it may be asymptomatic or misdiagnosed for a long time in a large proportion of patients [20, 21].

These unfavorable circumstances make interdisciplinary cooperation with all possible conservative treatment forms, including rehabilitation, the key to effective PAD treatment in patients with diabetes. The undertaken actions should be focused on how to slow the disease progression and stimulate the development of collateral circulation, which can cover the insufficient perfusion caused by the main vessel's trunk occlusion or its important stenosis.

This article is aimed at presenting the current place and possibilities of physiotherapeutic management in the course of PAD in patients with DM. This management is dedicated to patients with claudication and those with critical limb ischemia that can and cannot be operated on. Interventions are multistep and lifelong, just as the disease is chronic. They are often highly individualized due to multiple comorbidities that may, like some used medications, limit some forms of physiotherapy or necessitate their modification.

Rehabilitation consists of a number of consecutive but overlapping stages: assessment of the clinical condition at baseline and during the intervention, physiotherapy (individually tailored to the patient's condition and capacity), optimization of pharmacological treatment, control of modifiable risk factors (e.g., smoking cessation, education on healthy nutrition), foot care, education of patients and their families, and psychotherapy [22–25]. It requires close cooperation between the patient and the treatment team composed of an angiologist, diabetologist, vascular surgeon, physiotherapist, nurse, dietician, psychotherapist, and often a prosthetist.

## 2. Kinesitherapy

Kinesitherapy is recommended as the first-line treatment for PAD and T2DM, playing a therapeutic role in both diseases. This role comes down to a beneficial modification of known cardiovascular risk factors that can be influenced by the physical effort and almost immediately found, including hypertension, lipid disorders, obesity, and carbohydrates disorders. Additionally, patients benefit from exercise in many ways, often not immediately perceived, such as reduction of inflammation (underlying atherosclerotic process and aggravated in carbohydrate metabolism disorders) or increase in nitric oxide release, the most important vasodilator substance in the human body [12, 26, 27]. However, the patient with diabetes requires special involvement at the stage of exercise planning.

### 2.1. Planning Physical Effort in Peripheral Artery Disease in Patients with Type II Diabetes Mellitus

Conservative treatment in patients with DM complicated by PAD does not significantly differ from the recommended one in the population without DM; however, special emphasis is placed on glycemic control and the resulting limitations.

American Diabetes Association (ADA) guidance for individuals with DM focuses on glucose control with recommended hemoglobin A1c (HbA1c) < 7%, or close to 6%, to reduce the risk of complications. Both hyperglycemia (>250–300 mg%), as well as hypoglycemia (<100 mg%), should be generally avoided, but mainly before the patient starts physical activity. It protects individuals from ketoacidosis or uncontrolled lowering of the glucose during and after the exercise [28]. Moderate to vigorous aerobic exercise (≥30 min, 5–7 days/week) combined with anaerobic exercise (≥3 ×/week) is recommended to achieve better glycemic control. [29]. It affects both the modification of glycemic values [30] (both types of effort) and is a part of walking training (the aerobic one). Regular exercise modifies insulin effect in the muscles and liver. Aerobic exercise increases muscle glucose uptake up to fivefold in an insulin-independent manner. After physical training, glucose uptake remains elevated in insulin-independent (∼2 h) and insulin-dependent (up to 48 h) mechanisms if exercise is prolonged (48), which is linked with muscle glycogen pool restoration [31, 32]. On the one hand, this is a desirable effect, but on the other hand, it can promote hypoglycemia even hours after the activity cessation in some situations. Depending on the diabetes treatment, it is, therefore, essential to discuss the risk of hypoglycemia with the patient, as this is especially the case in patients treated with insulin or sulfonylurea [29]. In this issue, the contact between the team's members is critical because the lack of understanding of the mechanisms responsible for hypoglycemia may discourage patients from participating in exercise therapy from the very beginning.

Before physical training, individuals with DM should be screened for cardiovascular complications. It is part of safety features and arises from the common coexistence of the atheromatic lesions within other locations, mainly coronary circulation, together with the possibility of the autonomic neuropathy responsible for the so-called silent ischemia. Some medications (e.g., beta-blockers), often used for comorbid conditions, such as heart disease or hypertension, may limit exercise capacity through their effects on heart rate; other medicines can increase the risk of local bleeding (antiplatelet or anticoagulant medications) after possible injury, which should be monitored and information provided to the patients and their doctors.

Also, assessment of other chronic microvascular complications, including diabetic retinopathy, diabetic nephropathy, and peripheral neuropathy, may translate into recommendations for physical activity for patients with diabetes in general and those with coexisting limb ischemia.

According to the recommendations, when planning vascular rehabilitation in patients with DM, a typical blood circulation assessment (physical examination, additional tests) is necessary. Estimation of the claudication distance (initial and absolute) should be performed on a treadmill or, in case of limited possibilities, as a corridor test [29, 33]. Performing the test informs not only therapists of the clinical severity of the disease but also serves as an introduction to walking training education. It is also often used to assess the objective parameter of the postexercise ankle-brachial index (ABI) [33]. It should be taken into account that the sensitivity of resting ABI values is much lower in patients with DM [12] than in the general population. It is associated with an increased risk of arterial stiffness and microcirculation abnormalities [34]. If vascular sclerosis is not confirmed, ischemia occurs when the index value is ≤0.9. The progressively lowering value is associated with greater functional limitation and severe ischemia diagnosis when ABI is below 0.4–0.5 [33].

Mehta et al. [35] presented in 2020 the advantages and drawbacks of postexercise ABI based on a clinical case. The authors describe the mismatch between the recommended criteria for an abnormal postexercise ABI value and a low sensitivity of postexercise ankle systolic pressure decrease of more than 30 mmHg for diagnosing PAD. The authors suggest that postexercise ABI decline of more than 20% or postexercise ankle pressure decrease of more than 30 mmHg can establish PAD diagnosis and should prompt initiation of appropriate medical management [35].

In addition, to the posttest ABI assessment, if resting ABI is not possible to be interpreted, supplemental physiological testing studies may be indicated, including toe-brachial index (TBI), skin perfusion pressure, or transcutaneous oxygen pressure [33].

The exercise tests are also performed to assess and monitor the result of conservative treatment, e.g., six months into the rehabilitation program and the vascular procedure effectiveness [18].

### 2.2. Supervised Exercise Walking Training (SET)

The effect of diabetes on daily physical activity levels in patients with PAD is poorly studied, and current guidelines do not specify individual rehabilitation management recommendations for patients with PAD and diabetes. A recent study [36] reported that patients with DM and intermittent claudication presented less physical activity and reduced their physical function more than those with PAD alone. It has been suggested that diabetes contributes to reduced blood volume expansion and impaired skeletal muscle oxygenation. Because physical activity can correct these above disturbances [37], patients with DM may benefit from physiotherapy even more than the general population. A systematic review published in 2017 by Hageman et al. [38] summarized the literature dedicated to the impact of diabetes, as a comorbid disease, in patients with intermittent claudication (IC) on the effects of supervised exercise training. The review included randomized and nonrandomized studies with walking abilities, after SET, assessment in patients at 2nd level of the Fontaine's score. Considered outcome measures were maximal (absolute), pain-free (initial), and functional walking distance (MWD, PFWD, and FWD). The vast majority of the studies have confirmed improved walking performance in both patients with DM and patients without DM. Given the knowledge currently available, supervised interval treadmill training has a well-established role as a first-line therapy in the treatment program for patients with claudication [39, 40], including those with diabetes [12]. Improvements can be expected in both: PFWD (by 128 meters on average) and MWD (by 180 meters on average) [41]. The principles of supervised exercise, based on the 2007 TASC II guidelines [28], are regulated and updated by the European Society of Cardiology (ESC) guidelines prepared in collaboration with the European Society for Vascular Surgery (ESVS) [42]. According to the recommendations, exercise session should last from 30–60 minutes with rest breaks, just before the absolute distance of claudication reaching (the patient marches on moderate pain—so-called submaximal effort) [28, 33, 40, 42]. After walking cessation, it should be resumed once the pain subsides, which usually takes no more than 5 minutes. Sessions should be conducted 3–5 times per week for a minimum of 3–6 months [28, 33, 40, 42, 43], but generally should a lifelong procedure. Explaining the principles of this activity to the patient, including the acceptance of experiencing slight pain while walking, is essential for both the patient's psychological well-being and treatment outcomes [20]. Patient acceptance of the pain while walking with the understanding of its nonharmful character is crucial for proper implementation of the rehabilitation program but often difficult to achieve. It is because patients perceive ischemic pain as a contraindication to continuing exercise, extrapolating this situation from the rules surrounding physical activity in heart disease. The risk of coexisting neuropathy (including hypoesthesia) makes education about hygiene and proper footwear a unique element of the rehabilitation process. After each session, the patients should carefully examine the feet (on their own or with the help of another person) in order to detect injuries, calluses, abrasions, or areas indicating mismatched footwear (e.g., redness). The patients should remember to drink enough fluids during exercise to avoid overheating and dehydration [44–47].

In summary, SET is considered the gold standard regardless of the coexistence of DM but requires special attention and awareness due to more limitations resulting from this additional disease.

### 2.3. Nordic Pole Walking Exercise

In recent years, Nordic pole walking (NPW) has become a new, popular, and recommended form of training for cardiovascular disease patients. This technique engages arms and trunk muscles, significantly relieving the load on the lower limbs during walking [48, 49]. The beneficial effect of NPW on extending the distance of claudication was demonstrated in a study by Oakley et al. [50]. The authors emphasized its additional qualities as NPW might also be a helpful exercise strategy for improving the cardiovascular fitness of patients with intermittent claudication. It is of great importance as cardiac complications are the leading cause of death in this group [51]. Other reports on regular training with NPW in patients with PAD have confirmed that NPW training generally improved exercise tolerance, perceived quality of life (QoL), and decreased symptoms of claudication pain during walking [52–54]. Bulinska et al. [55] compared the efficacy of NPW training with traditional treadmill training on PFWD and MWD in patients with PAD. Rehabilitation sessions were performed three times per week for three months. The authors show that the MWD increased significantly more after an NPW program compared to walking without poles. Thus, studies confirm that NPW is at least as effective as traditional walking training when used in the rehabilitation of patients with PAD [55]. Additionally, the significant amount of muscles involved during this activity may also promote a better glycemic effect, and the method of performing this exercise makes patients feel much more secure.

### 2.4. Cycloergometer Exercise

Exercise on a bicycle ergometer is an excellent alternative for patients with DM complicated by PAD, to whom treadmill walking exercises are particularly uncomfortable or difficult [47]. This type of bicycle does not overload limb joints and spine as much as marching, which is favorably perceived by exercise participants, especially the elderly and obese. In addition, stationary cycling is beneficial for safety reasons (e.g., dizziness) and makes exercise independent of the weather. Before training on a bicycle ergometer, the patient should be instructed individually on the appropriate exercise load and the rules for performing the exercise. The patient should rest the front part of the foot on the bicycle pedal, which allows for a greater load on the distal limb muscles. The exercise should be performed daily, and the distance covered by the patient should be about 10 km [56]. The study conducted by Haga et al. evaluated the effectiveness of a three-month supervised bicycle program in improving walking abilities in patients with PAD. Intermittent claudication showed that bicycle exercise improved the MWD and QoL in patients with PAD. This training engages much more distal, below-knee muscles than the proximal ones, which is especially beneficial for patients with DM due to more common peripheral vessel stenosis in this group. The results showed that bicycling might be as useful as walking in patients with PAD [57].

### 2.5. Resistance Training

Current guidelines recommend resistance, anaerobic exercise as complementary training for patients with DM [33]. Dynamic resistance exercise, in particular, should be recommended for physically inactive individuals with chronic comorbid conditions like obesity, arthritis, and balance disorders, as they make it difficult to use other abovementioned traditional training. Resistance exercise increases limb muscle mass and strength [58] which translates into improved glucose metabolism. In addition, greater muscle mass promotes better balance and is a prerequisite for proper aerobic exercise, where symmetrical muscle work is required. However, this symmetry is often disturbed by sarcopenia, which results from both ischemia and involuntary relieving of the affected limb. Rebuilding muscle mass during the first months of therapy is, therefore, a necessary element of rehabilitation in this group of patients. Resistance training is well tolerated because, usually, it does not cause chronic pain. The ESC guidelines [12] emphasize that the combination of aerobic and anaerobic training is the most beneficial form of exercise for people with DM because of its strong, complex modifying effect on atherosclerotic risk factors such as hyperglycemia, lipid disturbances, and hypercoagulability, which characterize people with metabolic disturbances typical for insulin resistance. It is recommended that resistance training should be performed for approximately 15 minutes/3 days per week at 30–50% of maximum muscle strength followed by the rest. The maximum load should be determined before the training session. Exercises to improve flexibility and balance should be added to the above training forms, especially in patients with DM [12].

The review conducted by Machado et al. assessed the effects of combined aerobic and resistance exercise programs compared to the isolated aerobic exercise and the usual care in patients with intermittent claudication on walking performance [59]. Improvement was noted, as combined exercise and isolated aerobic exercise improved the claudication PFWD from 11 to 396% and 30 to 422%, respectively, and the absolute claudication distance from 81 to 197% and 53 to 121%. The authors emphasize that there is a strong need for randomized controlled trials to refine the strength of the effect of combining these activities in patients with PAD.

Despite the paucity of evidence regarding the effects of combining marching training with resistance training for patients with diabetes mellitus complicated by PAD, proposing combined exercise to this group of patients appears to be a reasonable and safe strategy to improve gait performance and modify cardiovascular risk factors in view of the basic knowledge relating to their mechanisms of action.

### 2.6. Particular Situations

In the aortic-iliac or femoral type of ischemia, patients can be additionally encouraged to perform exercises according to Horodynski's scheme (exercises of thigh and buttock muscles, e.g., squats). In the peripheral type of ischemia, Ratschow's and Buerger's exercises are recommended. These exercises are performed in the supine position with the provocation of extremity ischemia through extremity elevation, followed by forced passive congestion by lowering the lower limbs—similar rules are observed in upper extremity ischemia [60–62]. In patients at any stage of ischemia, including critical cases, which is a contraindication for walking training, the patients should be encouraged to perform individual rehabilitation program (e.g. respiratory, antithrombiotic, or/and upper extremity exercises). Patients with diabetes and PAD who cannot participate in supervised exercise sessions for many reasons should be advised to perform home-based exercise training (HBET) [47]. Collins et al. were the first to confirm the effectiveness of walking training, performed at home, in patients with DM complicated by PAD. In these patients, improvements were noted in walking distance, gait speed, and quality of life [63]. According to the recommendations, HBET should be done 3 to 5 times a week, from 15 (initially) to 40–50 minutes. Unsupervised walking training requires patients to be adequately trained on the safety and correctness of performing the exercise. This guarantees the effectiveness of the exercise. Walking should be dynamic, rhythmic, and performed on a flat, moderately hard surface (e.g., park paths) following the principles of personal safety and exercise hygiene like during supervised sessions. Despite the high efficacy of unsupervised training in well-motivated patients, its effectiveness depends on systematic reeducation of the patients and its periodical monitoring with the necessary correction made by the specialist. Many authors suggest that effective home-based exercise programs for individuals suffering from diabetes complicated with PAD may require ongoing contact with a physiotherapist, at least during the first six months [24, 47, 56, 64], to consolidate good habits in patients and to confirm how to perform marching exercises well. A study published in April 2021 by McDermott et al. identified the need for home-based exercise in patients with PAD and demonstrated the higher effectiveness of high intensity of these exercises [65]. The effectiveness of home exercise therapy was also confirmed in a study by Fukaya et al. [66]. The role of home-based exercise is emphasized in situations such as the current pandemic, where lockdowns have significantly reduced the use of structured forms of rehabilitation.

It should be stressed that rehabilitation is also an introduction (so-called prehabilitation) [67] and a continuation of surgical treatment. It should be good practice to admit patients to the surgical ward 2–3 days prior to vascular surgery for laboratory procedure and enable their education in postoperative rehabilitation. According to the 2019 guidelines on peripheral arterial disease by the European Journal of Vascular Medicine, rehabilitation should be implemented from the 14th day of discharge after the surgery [39]. Pandey et al. in 2017 [68] published an interesting meta-analysis of randomized, controlled trials to show the efficacy of initial endovascular treatment with and without supervised exercise training in patients with claudication. 7Change in PFWD, MWD, resting ABI was recorded to assess the risk of revascularization or amputations. First of all, the results underlined a known and obvious fact that endovascular procedure reinforced with supervised walking training was more effective than SET alone (improvement in total MWD, ABI, and reduction in the risk of revascularization or amputation). However, even more interesting was another analysis, which revealed that the use of invasive methods alone without complementary rehabilitation does not produce the expected improvement in functional capacity [68]. Thus, finally, the study stressed the role and strength of comprehensive treatment applied together.

## 3. Contraindications for Kinesitherapy

Exercise should be considered a part of the therapeutic management of patients with DM complicated by PAD. However, there are exceptional situations where they must be restricted. Exercise is contraindicated in patients with acute coronary heart disease until the patient's condition stabilizes, which takes approximately five days [69]. Other important exercising contraindications include advanced heart failure and rest dyspnea of any origin, recent myocardial infarction or active signs of ischemia in electrocardiogram (unstable angina), complete heart block, myocarditis, endocarditis, pericarditis, left ventricular outflow tract obstruction, pleuritis, pericarditis, and if systolic blood pressure falls by 30% of baseline during exercise. When blood pressure exceeds >160/100 mmHg, the exercise should be periodically discontinued [69–71]. Contraindications are also common in rheumatoid arthritis and osteoarthritis when there is acute inflammation or pain during exercising. There are several considerations that are important and specific for patients with diabetes. As was mentioned, significant hyperglycemia, mainly with a positive urine test for ketones and too low glucose (in patients treated with insulin or sulfonylureas), constitutes a contraindication [29]. Also, chronic diabetic complications like proliferative retinopathy and/or nephropathy should be considered an indication for an individualized approach. A condition that is specific to diabetes and requires special surveillance is the presence of abnormalities (deformities, abnormal location of the pressure on the sole) found on the patient's feet, as they promote the generation of local ulcers [45]. Providing the patient with special pressure-relieving insoles or special shoes and choosing exercises that reduce the chance of ulceration in the so-called high-risk foot (e.g., a cycloergometer with modification of the plantar support point on the pedal) gives a chance for safe and satisfying exercise.

## 4. How Does It Work? The Molecular and Biochemical Aspect of Exercise Training Offered to Patients with Pad Related to Diabetes Mellitus

Currently, despite the passage of many years from the introduction of the first principles of treatment for lower extremities atherosclerosis and despite significant advances in vascular surgery, physical activity remains an indispensable tool for improving limb blood supply [63]. It is achieved by activating many beneficial mechanisms, often not fully recognized, and modifying the most common risk factors that cannot be obtained with a surgical procedure [72–75]. The most important clinical effect is the improvement in claudication distance, but how do such beneficial changes occur? Vascular obstruction causes metabolic dysfunction at the skeletal muscle level. Chronic ischemia, along with the low level of physical activity, changes the phenotype in patients with PAD and results in decreased muscle tissue density, increases fat content, accelerates muscles cells apoptosis, reduces type I fibers, and reduces capillary density [72].

Many authors have shown that repetitive exercise improves hemorheology, thereby facilitating oxygen delivery to ischemic skeletal muscles [76–78]. It also improves the metabolism of the skeletal muscles and provides more economic mitochondrial energy production [72]. Exercise training improves oxygen extraction and carnitine metabolism within the working muscles also [79]. The upregulation of endothelial nitric oxide synthase, which improves nitric oxide release, is stimulated by increased shear forces during the excessive blood flow in muscles when working out. This could explain vasodilatation [80–82].

This was proved as exercise decreased systemic markers of inflammation, including mentioned molecules [72], and ultimately improved endothelial function [73, 83].

## 5. Other Therapeutic Tools

### 5.1. Pulsatile Pneumatic Compression Therapy

Intermittent/pulsatile pneumatic compression (IPC/PPC) appears to be a helpful adjunctive form of treatment for patients with PAD, including those with diabetes [84, 85]. Current findings related to this therapy indicate measurable effects in specific groups of patients [86]. IPC is based on transferring external pressure to the extremities by means of a pump that is periodically inflated with air or water. Pulsatile manner means that gradual compression applied to the legs to pressure value, usually close to the patient's systemic, diastolic one, is followed by the rapid deflation of the cuff. The compression result can be compared to the effect of fast walking, with no risk of pain or injury [87]. The procedure should be performed as often as possible (preferably daily) for about 2.5 h [62]. During the session, an increased arteriovenous pressure gradient reverses vasomotor paralysis, which secondarily increases nitric oxide release from endothelial cells, thus increasing vasodilation. Finally, perfusion is improved and, what is also very important, tissue edema can be reduced. Swelling often accompanies rest pain and results from patients holding their legs down for many hours to resolve the pain, but unfortunately causing constriction of small, mainly subcutaneous blood vessels that exacerbate the ischemia. For patients who are ineligible for surgical intervention due to contraindications and/or lack of technical feasibility, intermittent compression therapy may be associated with improved amputation-free time survival, less rest pain, and improved quality of life [87].

Alvarez et al. [88] evaluated the efficacy of treatment with high pressure (IPC) in subjects with symptomatic PAD or CLI symptoms.

It has been shown that this type of IPC promotes the healing of wounds and reduces associated chronic pain in subjects with CLI and improving walking distance in patients with intermittent claudiction (IC). The authors suggest that IPC is safe and effective and should be considered for patients who are not candidates for endovascular or surgical procedures.

IPC is a safe therapy that can be used both in hospital or outpatient conditions and, after appropriate patient training, also at home [86]. The use of the so-called circulation boot, which is a variation of the “soft” form of IPC, gives the opportunity of topical application of antibiotics or other substances improving ulcer healing [89, 90].

### 5.2. Physical Therapy

Physical therapy is recommended as adjunctive or the only treatment when there are contraindications for other methods [56, 91]. Patients with good glycemic control, without acidosis, and who do not present severe vascular complications can be qualified for physical therapy [91, 92]. The treatment effectiveness is determined by the mechanisms expected to lead to vasodilation and can present an analgesic and/or anti-inflammatory effect. The effect of physical stimuli on the improvement of tissue metabolism, peripheral nerve, and vascular function can help treat ulcers in patients with diabetic foot syndrome. Often, additional effects are achieved during treatment, including improvement in complaints resulting from comorbidities (e.g., osteoarthritis), which not only affects the patient's positive perception of the treatment but also helps to prepare the patient more effectively for physical exercise by reducing pain from causes other than ischemia [41, 56, 91, 93].

Physical therapy agents most commonly used to treat patients with DM and PAD include phototherapy with low-energy lasers and polarized light; magnetotherapy; electrotherapy with TENS currents, iontophoresis, longitudinal galvanization, diadynamic currents, electrostimulation; ultrasound; and heat treatments (local and general), short-wave diathermy, and shock wave [56, 91–96].

However, it is worth noting that due to quite frequent sensory failure present in diabetes, the application of currents or thermal treatments should be carefully considered to avoid burns [56].

Studies conducted so far [92, 96–101] have demonstrated the effectiveness of the abovementioned physical therapies in reducing the extent of ischemic foot ulcers and improving limb blood supply [92, 97–101] in individuals with DM.

However, it should be noted that not all studies so conclusively support the validity of physical therapy. In their multicenter, double-blind, randomized controlled trial, Ennis et al. did not confirm the effectiveness of ultrasound treatment on wound healing in patients with chronic diabetic foot ulcers [102]. Also, in a randomized crossover study conducted by Guirro et al., no statistical significance was found to support the short-wave diathermy treatment in a group of patients with a similar condition [96].

Of note, the role of complex therapy, understood as physiotherapy, is carried out in spa conditions. In addition to the possible coapplication of different types of supervised physical activity, behavioral education, and physical therapy procedures, the use of typical spa forms of therapy may be an additional benefit (e.g., mood therapy, water-based therapy). In patients with DM and chronic circulatory complications, health-resort treatment directed at improving blood supply to the tissues is recommended. Acid-carbonated water and gas baths, sulfide-sulfide baths, and whirlpool massage of the lower limbs increase skin flow and dilate blood vessels [94]. Mud paste packs, which dilate capillary vessels, are also used. A combination of the above agents can also be applied [103].

However, it should be emphasized that the current studies on the effectiveness of physical therapy methods and accompanying economic aspects are not exhaustive. Nevertheless, physical treatments and spa therapies seem to be an attractive alternative or supplement to the standard therapy for selected individuals, especially those with no invasive treatment options [56].

### 5.3. Systemic Hyperbaric Oxygen Therapy (HBOT)

The consistently unsatisfactory therapy results for critical limb ischemia have forced the search for adjunctive forms of treatment to surgery. One of the alternative or complementary methods is systemic hyperbaric oxygen therapy. Hyperbaric therapy involves placing the patient in a chamber where 100% oxygen is applied under increased pressure. The oxygen thus administered dissolves in the plasma, which allows its transport to the tissues with very poor perfusion. Hyperbaric oxygen therapy increases neutrophil killing ability, stimulates angiogenesis, and enhances fibroblast activity and collagen synthesis [104]. An absolute contraindication to the use of conjugated oxygen therapy is untreated pneumothorax and chemotherapy [105]. Data relating to the efficacy of HBOT are conflicting. While some authors do not indicate that this method significantly improves ischemic ulcer healing [106], others report the effectiveness of HBOT in treating the infected diabetic foot [104, 107, 108]. The last meta-analysis performed in January 2021 concludes that HBOT was associated with higher rates of diabetic foot ulcer healing and reduced rates of major amputation of the lower extremity. The authors suggest a great need for well-planned, sufficiently multicentric trials to assess the efficacy and safety of HBOT as an adjuvant treatment for diabetic foot ulcers [109].

In conclusion, HBOT is a rapidly growing branch of medicine. The use of this form of therapy to treat ischemic lesions in patients with diabetes requires further research. Nevertheless, HBOT should be considered an adjunctive or alternative part of treatment for patients with diabetic foot.

### 5.4. Lower Limb Offloading

In terms of physiotherapeutic management of patients with CLI, lower limb pressure relief is also indicated. It is recommended primarily for patients with neuropathic foot, but it also supports the treatment of ischemic ulcers. Among other things, weight-bearing helps to improve perfusion in the foot [28] and prevents damage to the foot by inappropriate footwear, especially if any deformation is present [110]. Different methods can achieve partial or complete pressure-relieving, i.e., shoe modification [111–113], individually tailored insole [114], podological treatment using individually tailored materials [110], also pelotics (protection of sensitive areas is achieved by absorption of pressure forces by a pressure-relieving material), taping [115], special plaster dressings [116, 117], but also use of crutches [118] or wheelchair in exceptional situations. The choice of the method to offload the pressure on the sole depends on the location of the ulceration and its severity but also the patient's general condition, ability, and acceptance.

Discussing each method of pressure relief is beyond the scope of this paper. It is important to recognize that methods such as using crutches or a wheelchair are also ways for individuals after amputation to get around in the first few months after surgery (sometimes by patient choice or necessity, for the rest of their lives). In both cases, the patient's mobility is determined by upper limb muscle strength. The use of electric wheelchairs is still not standard in many countries; thus, the patient's condition and upper extremity strength play a crucial role in an individual's independence. What is more, mobilizing the upper limb muscles improves overall patient function.

Technically, using a wheelchair is easier than walking on crutches. However, crutches allow the patient to get to places not available when using a wheelchair. The crutch phase, because of the difficulties encountered by the person, should be an important part of the rehabilitation process to prepare the patient for elective limb amputation [119, 120]. This elective procedure gives the medical team time to improve the patient's dynamic parameters of the limbs and trunk and thus build better performance of daily living activity after surgery. A significant effect of exercise should also maintain or even increase the joints' range of motion.

Bearing in mind the higher risk of critical limb ischemia with a higher rate of amputation in patients with diabetes, it is necessary to incorporate exercises of the shoulder girdle and free part of the upper extremity muscles in patients' rehabilitation programs to protect them from future disability.

## 6. Conclusions

Unfortunately, despite the proven and essential role of different forms of kinesitherapy in walking ability improvement, there is still too little knowledge about the impact of physical therapy, e.g., on wound healing or ischemic pain relief. Our faith in modern medicine procedures slowed down the studies in this field and uncovered many aspects that require further research. With the growing number of comorbidities, including diabetes and atheromatosis, the aging population makes rehabilitation the key to successful and optional treatment. The population cannot always be treated with the usual treatment options, and the recommendations for rehabilitation based on evidence medicine are strongly needed. However, the results of the available study give us the opportunity to develop comprehensive therapy, including rehabilitation.
